# Automated liver lesion detection in ^68^Ga DOTATATE PET/CT using a deep fully convolutional neural network

**DOI:** 10.1186/s13550-021-00839-x

**Published:** 2021-10-02

**Authors:** Jonathan Wehrend, Michael Silosky, Fuyong Xing, Bennett B. Chin

**Affiliations:** 1 Division of Nuclear Medicine and Molecular Imaging, Department of Radiology, University of Colorado School of Medicine Anschutz Medical Campus, 12401 East 17th Avenue, Mail Stop L954A, Aurora, CO 80045 USA; 2 Department of Biostatistics and Informatics Colorado School of Public Health, University of Colorado Anschutz Medical Campus, Aurora, CO USA

**Keywords:** Deep learning, Convolutional neural network, Neuroendocrine tumor, DOTATATE, Somatostatin receptor, Positron emission tomography, Liver tumor

## Abstract

**Background:**

Gastroenteropancreatic neuroendocrine tumors most commonly metastasize to the liver; however, high normal background ^68^Ga-DOTATATE activity and high image noise make metastatic lesions difficult to detect. The purpose of this study is to develop a rapid, automated and highly specific method to identify ^68^Ga-DOTATATE PET/CT hepatic lesions using a 2D U-Net convolutional neural network.

**Methods:**

A retrospective study of ^68^Ga-DOTATATE PET/CT patient studies (*n* = 125; 57 with ^68^Ga-DOTATATE hepatic lesions and 68 without) was evaluated. The dataset was randomly divided into 75 studies for the training set (36 abnormal, 39 normal), 25 for the validation set (11 abnormal, 14 normal) and 25 for the testing set (11 abnormal, 14 normal). Hepatic lesions were physician annotated using a modified PERCIST threshold, and boundary definition by gradient edge detection. The 2D U-Net was trained independently five times for 100,000 iterations using a linear combination of binary cross-entropy and dice losses with a stochastic gradient descent algorithm. Performance metrics included: positive predictive value (PPV), sensitivity, F_1_ score and area under the precision–recall curve (PR-AUC). Five different pixel area thresholds were used to filter noisy predictions.

**Results:**

A total of 233 lesions were annotated with each abnormal study containing a mean of 4 ± 2.75 lesions. A pixel filter of 20 produced the highest mean PPV 0.94 ± 0.01. A pixel filter of 5 produced the highest mean sensitivity 0.74 ± 0.02. The highest mean F_1_ score 0.79 ± 0.01 was produced with a 20 pixel filter. The highest mean PR-AUC 0.73 ± 0.03 was produced with a 15 pixel filter.

**Conclusion:**

Deep neural networks can automatically detect hepatic lesions in ^68^Ga-DOTATATE PET. Ongoing improvements in data annotation methods, increasing sample sizes and training methods are anticipated to further improve detection performance.

**Supplementary Information:**

The online version contains supplementary material available at 10.1186/s13550-021-00839-x.

## Background

Neuroendocrine tumors (NETs) are rare neoplasms that can present with a wide array of symptoms, frequently contributing to delayed diagnosis and late presentation with metastatic disease [1]. Early and accurate detection of NETs is important for appropriate treatment. Low- and intermediate-grade NETs frequently have high expression of somatostatin receptors (SSTR subtype 2), an ideal target for a radionuclide-bound peptide imaging and therapy [2, 3]. The risk of NET metastasis is greatest for tumors arising in the pancreas and small intestine, with the liver being the most common site for metastasis (82% of patients) [4].

^68^Ga-DOTATATE PET/CT has demonstrated the highest accuracy in detection and staging of gastroenteropancreatic neuroendocrine tumors (GEP-NETs) [5]), and high uptake is essential for effective ^177^Lu-DOTATATE peptide receptor radionuclide therapy [6]. Despite the treatment benefit of ^177^Lu-DOTATATE of improved progression-free survival, only 17% had a partial response and only 1% had a complete response. The majority of these patients had persistent disease, potentially requiring retreatment [7]. High pre-therapy ^68^Ga-DOTATATE tumor uptake, low ^18^FDG avidity, low tumor volume and a significant change between ^68^Ga-DOTATATE uptake pre- and post-therapy show some correlation with response to treatment and patient outcomes [8, 9]. Yet, despite the need for retreatment, no established method to quantitatively assess response to therapy currently exists. Although subjective ^68^Ga-DOTATATE PET/CT interpretation is relatively consistent [10], an objective method could greatly improve the assessment of therapy response and facilitate the development of next-generation therapies.

Recently, convolutional neural networks (CNNs) have demonstrated high accuracy in lesion classification, image segmentation and object detection tasks with FDG PET/ CT [11–16]. Previous studies have demonstrated excellent results in classification and localizing lesions using ^18^F-FDG PET/CT; however, no such CNN has been created to analyze studies utilizing ^68^Ga-DOTATATE PET. Development of deep learning algorithms to identify tumors could be a valuable diagnostic and prognostic tool by aiding readers in the detection and quantification of lesions, and by identifying residual tumors after therapy. The purpose of the study is to develop and test a deep learning algorithm to accurately detect ^68^Ga-DOTATATE avid hepatic lesions.

## Methods

### Patients

This study was approved by our Institutional Review Board, and informed consent was waived. Between 1/10/18 and 4/16/20, 290 ^68^Ga-DOTATATE PET/CTs were performed at our University hospital on conventional analog detector PET/CT (GE Discovery STE; GE Medical systems). Of the 290 studies performed and retrospectively reviewed, 106 were found to have ^68^Ga-DOTATATE avid hepatic lesions. Of these, 58 of the studies met the inclusion criteria of fewer than 10 well-defined, non-confluent lesions without liver disease that would significantly impact the interpretation of the study or result in poorly defined or abnormal background activity (i.e., cirrhosis or steatosis). These studies were paired with 68 ^68^Ga-DOTATATE PET/CT studies evaluating for metastatic NETs which were found to have no liver metastases.

### Manual liver segmentation and semi-automated lesion annotation

Prior to analysis, all studies were anonymized using a five-digit numerical ID. The ^68^Ga-DOTATATE PET/CTs were clinically reviewed and reported by three board-certified Nuclear Medicine physicians with > 5 years’ experience. Lesions identified by the resident performing the workflow were correlated with the experienced board-certified Nuclear Medicine attending physician’s clinical report. Segmentation was performed by two trained physicians using a semi-automated MIM workflow (MIM version 7.03) that integrated segmentation of the liver on PET and CT with the modified PERCIST criteria for lesion detection [17]. The modified PERCIST threshold is based on the normal background liver volume of interest, placed by the semi-automated workflow and modified by the physician operator, to provide mean and standard deviation of ^68^Ga-DOTATATE activity using three separate spheres (3 cm diameter) placed in normal background liver. The computer workflow would automatically generate the 3 cm spheres; however, they were manually placed and verified in the liver by the operator. The modified PERCIST threshold was defined as the mean normal activity multiplied by 1.5, plus 2 standard deviations of background normal liver. To ensure accurate and consistent identification of lesions for the gold standard, results of the semi-automated workflow were correlated with the studies’ original clinical report. Once lesions detected by the workflow were confirmed as correct, a commercially available gradient edge detection tool (PET Edge plus; MIM software 7.0.3) was used to define lesion boundaries. All lesions were then verified visually by the resident physician operator who has the option of accepting, modifying or deleting these based on visual inspection. The time required to annotate the lesions was highly dependent upon the number of lesions in the liver. On average, the entire manual annotation and segmentation process took approximately 30 min per study. Some individual processes were assisted by the semi-automated workflow, such as verifying correct lesion detection and boundaries.

Studies were transferred from the analysis workstation (MIM) to a secure remote server. A custom pipeline written in Python (version 3.7.6) converted the PET images and regions in RT-structure format into 8-bit PNG image files. The PET images were converted without alteration; however, the liver segmentation data and lesion annotations were converted into binary color maps for use within the CNN. The dataset was then randomly divided into 75 studies for the training set (36 abnormal, 39 normal), 25 for the validation set (11 abnormal, 14 normal) and 25 for the testing set (11 abnormal, 14 normal).

### CNN architecture, training and validation

**Fig. 1:**
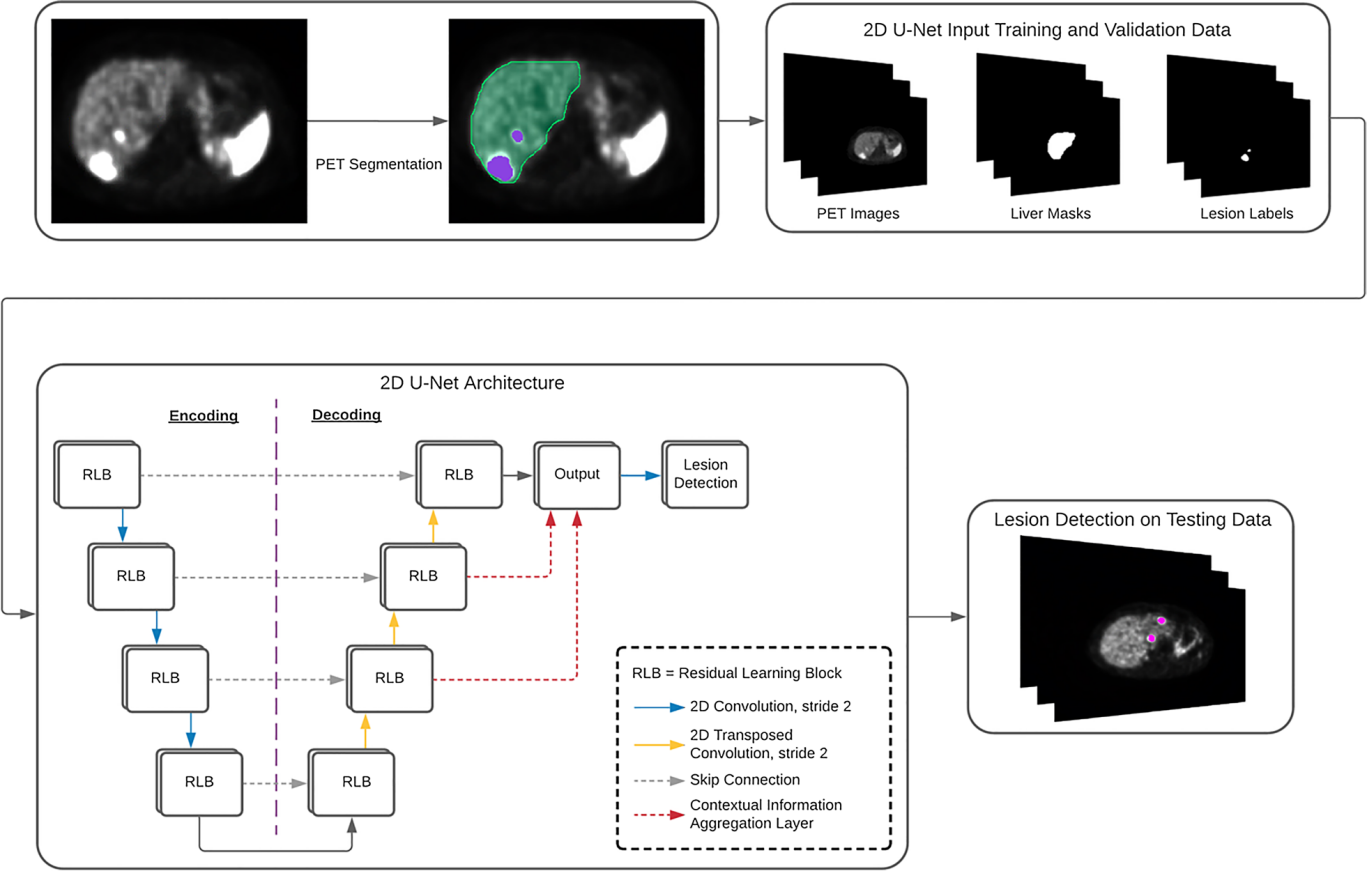
Overview of the convolutional neural network developed for automated detection of ^68^Ga-DOTATATE avid hepatic lesions. Each study in the training and validations sets contained three directories of images: PET images, liver masks and lesion labels. The CNN was trained on the masked PET images with lesion labels as ground truth. The trained network was then used for detection on the testing data, again with associated lesion labels used as the ground truth

The CNN developed for this study is a 2D fully convolutional, U-Net-like neural network. The encoder–decoder architecture contains a down-sampling path, which consists of four stacked residual learning blocks [18] and a convolutional operation with stride of 2 used to connect adjacent blocks. The up-sampling path also contains four residual blocks linked up with transposed convolutions with stride of 2 [19]. Four long-range skip connections were used to directly connect the outputs of the down-sampling residual blocks to the outputs of corresponding up-sampling residual blocks. We also introduced two contextual information aggregation layers to the up-sampling path and fused the information with the output of the last residual block, which is fed into a final convolutional layer for lesion detection. These two aggregation layers use transposed convolutions with stride 4 and 8, respectively. The 2D U-Net network was trained by minimizing a linear combination of binary cross-entropy loss and dice loss with a stochastic gradient descent algorithm for 100,000 iterations and stopped the training if the performance on the validation set did not improve for 20,000 successive iterations [20]. We applied the trained model to each slice in the test set for output map prediction. To reduce the effects of noisy predictions, filters based on pixel area were applied that removed values below a certain threshold (e.g., areas of 5, 7, 10, 15 and 20 pixels) to determine which generated the best results. We finally located true positive predictions if the intersection over union between the predictions and corresponding gold standards was greater than 0.05. Figure 1 details the flow of data and architecture of the 2D U-Net visually. Additional information on performance metrics was chosen, and loss functions and model implementation information is available in the Additional file 1.

### Statistical analysis

The performance of automated ^68^Ga-DOTATATE lesion detection was evaluated with two separate metrics: F_1_ score, the weighted means of positive predictive value (PPV) and sensitivity, as well as the area under the precision–recall curve (PR-AUC). Here, precision is the same as PPV and recall the same as sensitivity, but PR-AUC is retained as the more commonly used nomenclature. Once optimal hyperparameters for training the model were found, the training, validation and testing processes were independently run a total of five times to account for random variations in image augmentation during the training process and ensure that the model performed consistently. The mean of the five PR AUC and F_1_ scores was then taken. All statistical analysis was performed with R 4.0 (open source, GNU project).

## Results

**Fig. 2:**
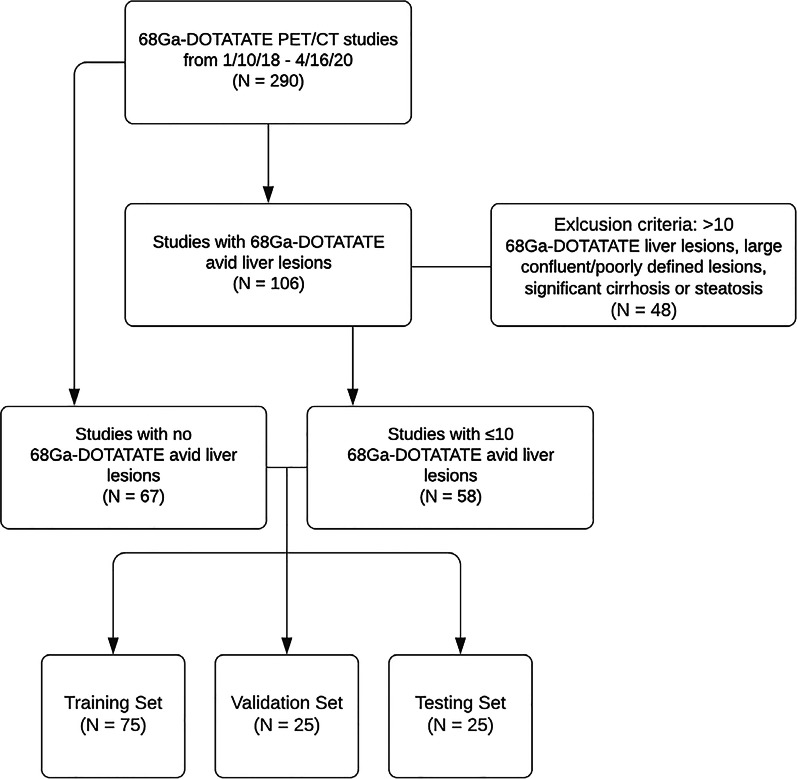
Study flowchart of subjects used in this study

A total of 125 patients with a mean age of 61.2 ± 13.4 met inclusion criteria and were included in this study. Of these, 68 subjects (54.4%) were female, and 57 (45.6%) were male. Metastatic NETs were present in 58 (46.4%) of the subjects, while 67 (53.6%) had no hepatic tumor burden. Of the 58 studies with ^68^Ga-DOTATATE avid hepatic lesions, 233 distinct lesions were annotated by two trained physicians and correlated with Nuclear Medicine fellowship trained clinical interpretations. There were 4.0 ± 2.75 (mean ± STD) lesions per abnormal study, with each lesion typically present on 4–5 consecutive slices and studies ranging between 23 and 71 trans-axial slices. Flow diagrams for the dataset can be found in Fig. 2 and patient demographics and baseline characteristics can be found in Table 1.

**Table 1:** Patient demographics and baseline characteristics

Parameter	Value
Mean age (years)	61.2 (13.4)
Women	60.2 (14.3)
Men	62.3 (12.4)
Sex (no. of patients)	
Women	68 (54.4)
Men	57 (45.6)
Tumor present in liver	
Yes	58 (46.4)
No	67 (53.6)
Primary tumor site	
Small bowel	53 (42.4)
Pancreas	28 (22.4)
Large Bowel	12 (9.6)
Retroperitoneal	5 (4.0)
Head and neck	4 (3.2)
Lung	3 (2.4)
Stomach	3 (2.4)
Esophagus	1 (0.8)
Gall bladder	1 (0.8)
Prostate	1 (0.8)
None (normal scans)	14 (11.2)
Ki-67 index	
Low/intermediate (< or = 20%)	66 (52.8)
High grade (> 20%)	3 (2.4)
No pathology report/Index not reported	56 (44.8)

Values in parentheses are percentages, except for age (Standard Deviation)

Of the various models tested on the dataset, a 2D U-Net using a 6:1 linear combination of binary cross-entropy and dices losses was found to produce the best and most consistent results. A lesion was considered to be detected if the intersection over union of the prediction and gold standard was > 0.05. For the five runs of the model, a pixel area noise filter was applied to the predicted lesions at various pixel area thresholds and any prediction below the threshold was discounted from the quantitative analysis. A noise filter of 5 pixels was found to produce the highest sensitivity (0.74 ± 0.02), whereas the highest PPV was produced with a filter of 20 pixels (0.95 ± 0.01). A noise filter of 20 pixels was also produced the highest mean F_1_ score (0.79 ± 0.01). For all above values, an F_1_ threshold of 0.05 was used. The threshold is distinct from the noise filter and was used to binarize the data in the prediction map, where any value above the threshold was considered a lesion as opposed to background. The F_1_ scores increased with increasing filter size, ranging from 0.66 ± 0.02 at 5 pixels to 0.79 ± 0.01 at 20 pixels. Similarly, precision increased with increasing filter size, whereas sensitivity decreased. The five PR AUC values exhibited slightly more variability, ranging from 0.70 to 0.73, with the highest value at a noise filter of 15 pixels (0.73 ± 0.03). While F_1_, PPV, sensitivity and PR-AUC varied between pixel filter thresholds used, the values were highly consistent between the five runs of the model with low standard deviations for all. The mean the metrics of the five retrained models at all tested pixel thresholds is shown in Table 2.

**Table 2:** Metrics used to track model performance on the testing data at different pixel noise threshold levels

Parameter (STD)	Noise filter threshold in pixels				
	5	7	10	15	20
PPV	0.69 (0.03)	0.75 (0.03)	0.82 (0.01)	0.90 (0.02)	0.94 (0.01)
Sensitivity	0.74 (0.02)	0.74 (0.03)	0.72 (0.02)	0.69 (0.02)	0.63 (0.03)
F_1_ score	0.66 (0.02)	0.68 (0.02)	0.72 (0.02)	0.76 (0.01)	0.79 (0.01)
PR AUC	0.70 (0.02)	0.71 (0.02)	0.72 (0.02)	0.73 (0.02)	0.71 (0.02)

All data points are the mean of the five retrained models. F_1_ is a weighted mean of PPV and sensitivity, giving a composite score for model performance. PR AUC is the area under the precision–recall curve, where precision is PPV and recall is sensitivity. Data in parentheses are the standard deviations of the mean of the five retrained models

**Fig. 3:**
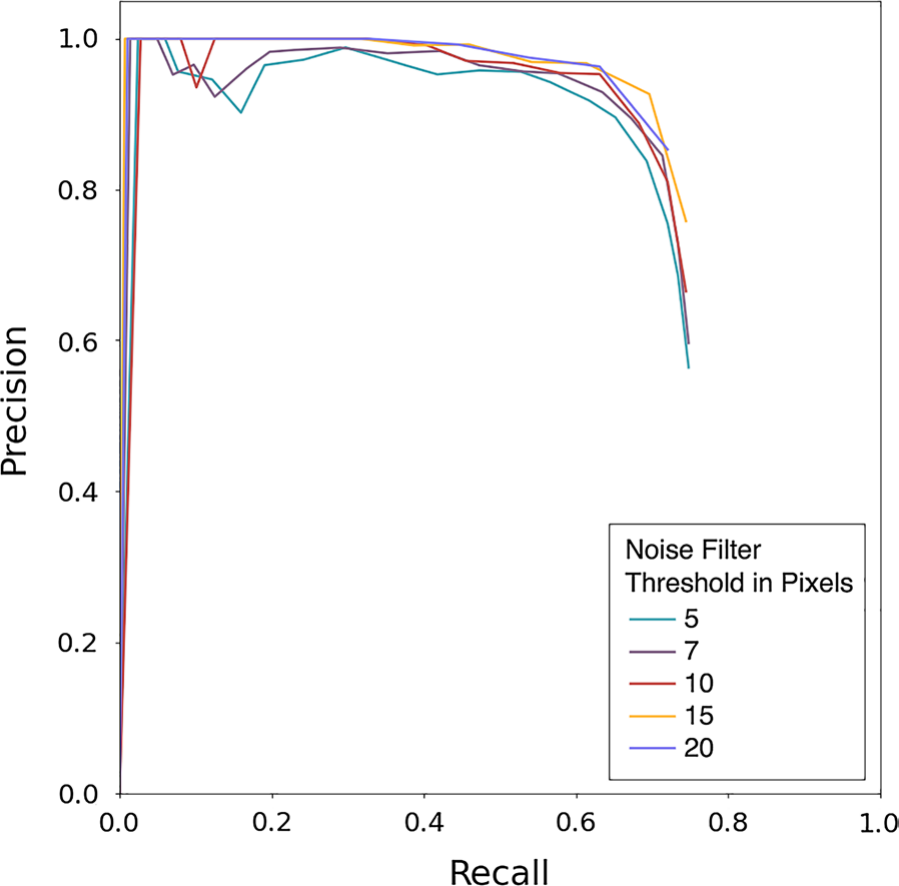
Precision–recall curves for the model’s evaluation on the testing dataset. Each curve represents one of the five different pixel area thresholds that were used in ^68^Ga-DOTATATE avid hepatic lesion detection. The greater the area under the curve (AUC), the better the model was at correctly predicting true lesions. Precision is equivalent to positive predictive value and recall is equivalent to sensitivity

**Fig. 4:**
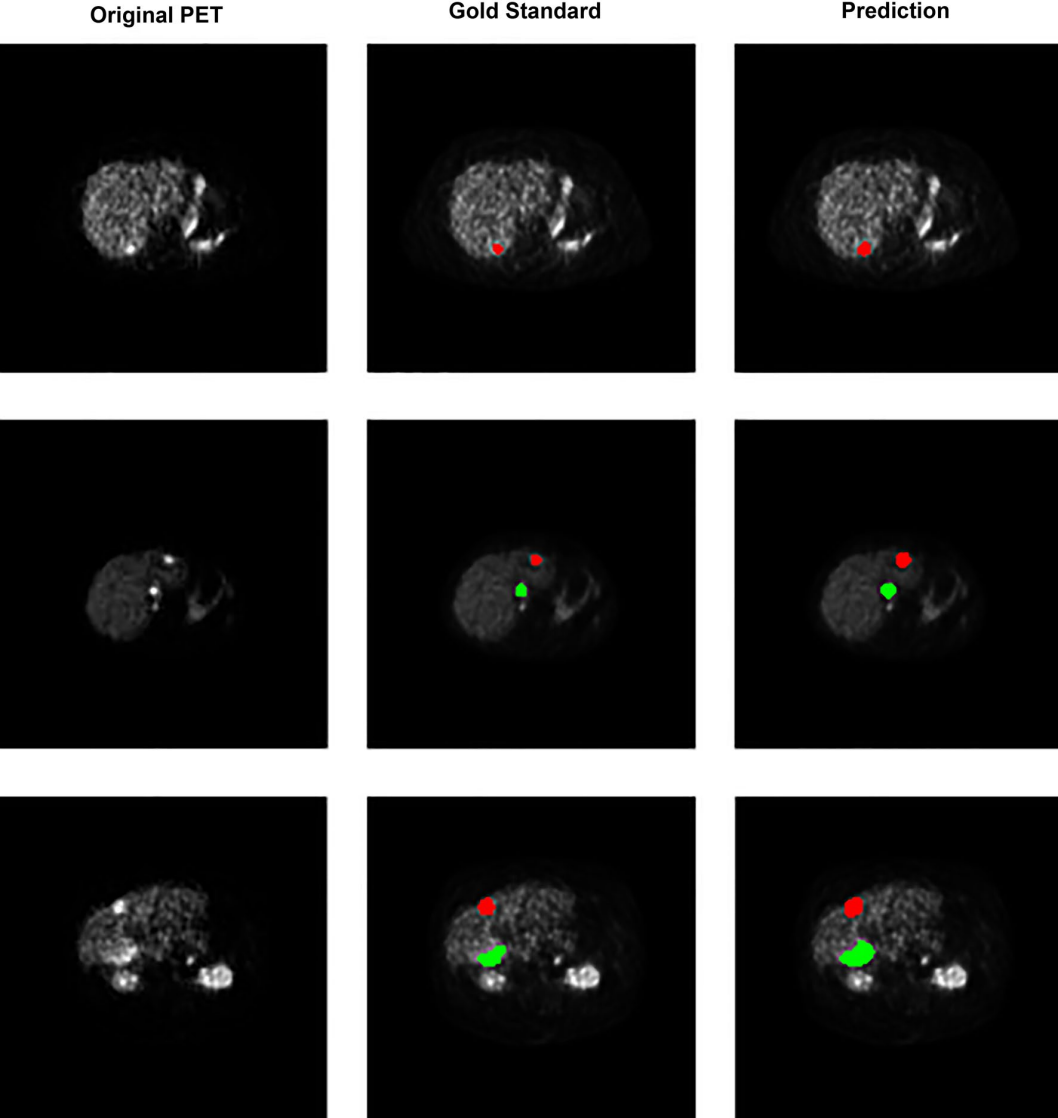
Output of the prediction model on the testing dataset showing *true positives*. Each row of transaxial PET slices is taken from a single study. (left column) original PET image, (middle column) physician-annotated ground truth and (right column) the output of the model prediction overlayed on the PET image

**Fig. 5:**
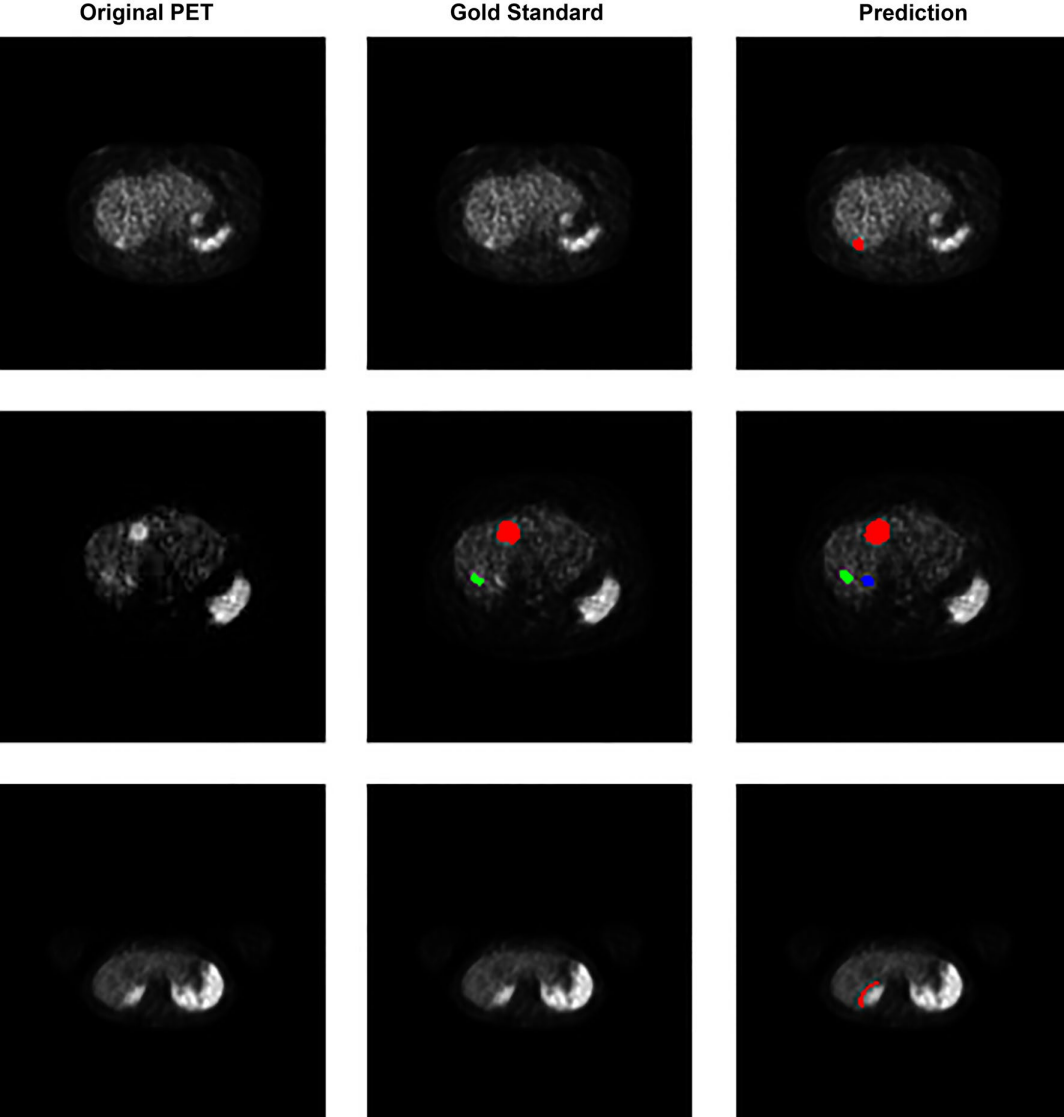
Output of the prediction model on the testing dataset showing *false positives*. The first and second patient rows demonstrate small voxel areas not detected using the gold standard criteria, but they were detected as lesions by the trained model. These “false positive” lesions are on transaxial slices immediately adjacent to “true positive” gold standard lesions (at the edge of a true positive). Row three demonstrates a false positive due to significant misregistration between the PET and CT. Each row of transaxial ^68^Ga-DOTATATE PET slices is taken from a single study

**Fig. 6:**
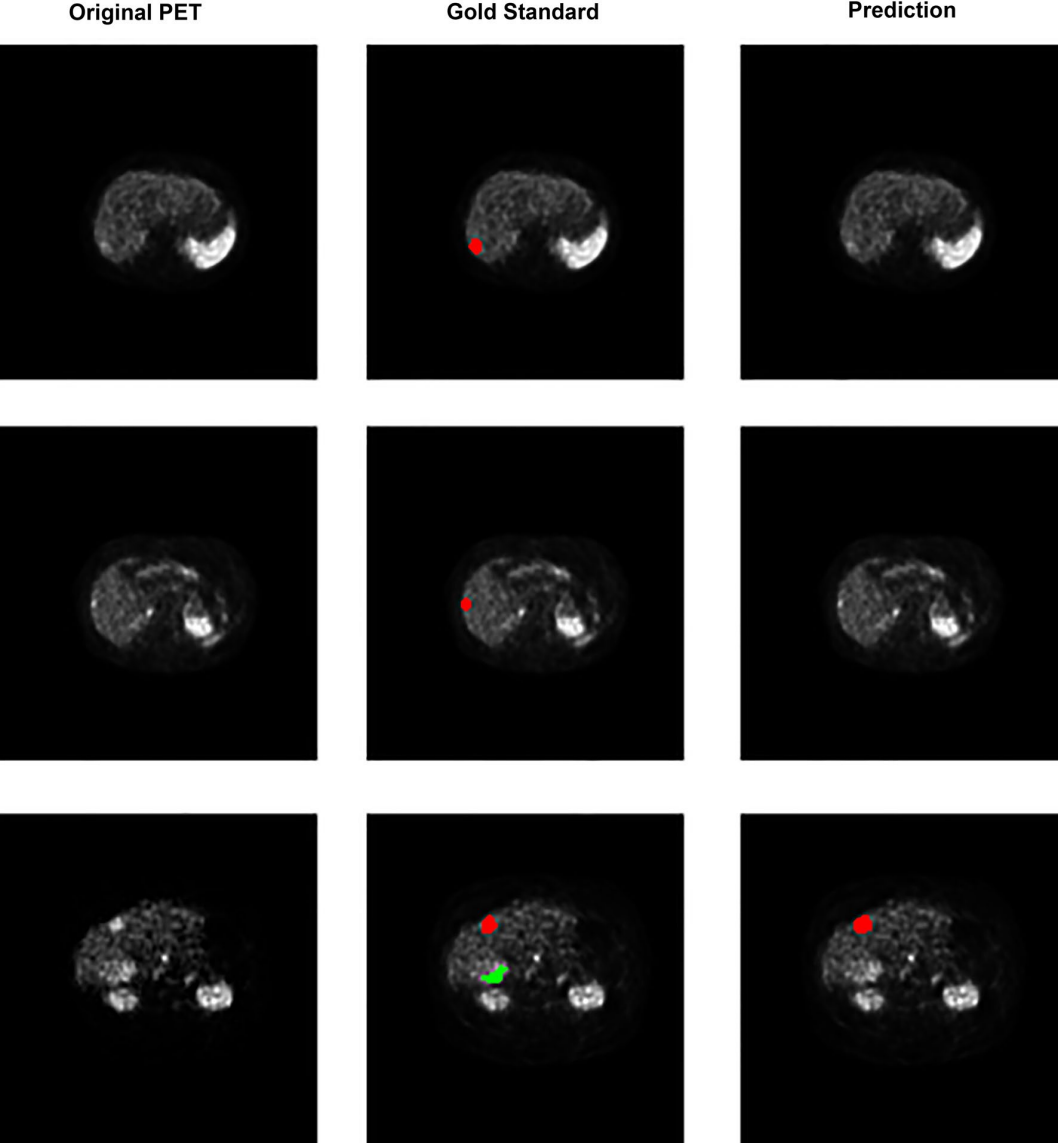
Output of prediction model on the testing dataset showing *false negatives*. These lesions were detected using the modified PERCIST, and gradient edge criteria, however, were not detected by the trained model on the testing data. The majority of these lesions were relatively low in activity. Each row of ^68^Ga-DOTATATE transaxial slices is taken from a single patient study

A graphical representation of the precision–recall curve is shown in Fig. 3. The various filter sizes performed relatively similarly for PPV and sensitivity, with nearly all filter permutations decreasing rapidly at a sensitivity of approximately 0.8. An example of the model is shown in Fig. 4, which shows true positives as output by the model. The slices demonstrate close approximation between the physician-annotated lesion labels and lesion labels output by the trained model. Figure 5 shows examples of model output false positives. A common false positive was due to a small number of voxels with high uptake located on a transaxial slice that was adjacent to a true positive lesion. False negatives are shown in Fig. 6 where a lesion was present on the annotated dataset but not detected by the trained model, adjacent to a true positive.

## Discussion

This deep learning-based, automated hepatic lesion algorithm for ^68^Ga-DOTATATE PET consistently performed with good F1 scores when noise filtering was applied to the prediction modeling. The use of a noise filter (15 voxels) provided the highest precision recall AUC 0.73 ± 0.02, which was also associated with an acceptable F1 score 0.76 ± 0.02, an excellent PPV 0.90 ± 0.02 and a modest sensitivity 0.69 ± 0.02. A semi-automated method of data annotation (modified PERCIST and gradient edge) provided a rapid method to identify lesions and determine the lesion boundaries with minimal user intervention. The results reported are concordant with expected values based on the relatively modest sample size, the inherent difficulty of the lesion identification task and the relatively noisy images compared to FDG PET. Future developments extended to whole body analysis in larger studies could add to the growing body of evidence that deep learning analysis of PET/CT is capable of predicting response to treatment and survival similar to data emerging for FDG PET/CT [21, 22].

This study uses a semi-automated method to assist the physician operator in defining the lesions and their boundaries for the annotated dataset used to develop the algorithm. Manual data annotation is extremely time and effort intensive and can be a limiting factor in obtaining adequate datasets for deep learning algorithms [23]. In preliminary work, the use of a modified PERCIST to both identify the candidate lesion and to define their boundaries resulted in relatively modest performance yielding an initial F_1_ score of 0.60 [17]. By modifying the boundary definition using a gradient edge detection method and using a pixel noise filter, the F_1_ metric improved to as high as 0.79. This method demonstrates the potential for better DL algorithm performance when the criteria for lesion boundaries more accurately depicts the true lesion boundary.

This method also allows flexibility in defining how sensitive the input data can be to define a true lesion. Because we set this threshold to a relatively high level, it facilitates a rapid (approximately 3 min per subject), semi-automated editing and approval by the physician who is annotating the data. A lower threshold could be chosen to result in higher algorithm sensitivity at the cost specificity; however, it potentially requires more time and effort by the physician when editing the annotations. The current data annotation method demonstrates the potential to rapidly generate high-quality annotated datasets while simultaneously reducing the time and effort required by a skilled physician operator. Finally, a semi-automated method could improve consistency and reduce operator bias. After further validation, the rules for lesion identification and boundary definition could be more readily adapted by other users, thereby facilitating a mechanism to acquire data from multiple institutions and facilitate a federated model of deep learning.

The study task and the PET imaging data in this study are also quite different compared to prior studies of FDG PET. To our knowledge, this is the first study to report automated hepatic lesion detection using deep learning in ^68^Ga-DOTATATE PET. Hepatic lesion detectability is an especially difficult task in ^68^Ga-DOTATATE imaging because of high normal background liver activity [5, 24] and high variability of uptake [24–27]. Prior studies of ^68^Ga-DOTATATE PET have confirmed that detectability is not only dependent on tumor uptake, but it is also highly dependent upon the normal background and the background image noise [28, 29]. These factors highly influence the signal-to-noise ratio (SNR), a metric of lesion detectability [28].

^68^Ga-DOTATATE PET lesion detectability is also influenced by higher image noise compared to FDG PET due to lower administered dose and rapid radionuclide decay. The ^68^Ga-DOTATATE administered dose (typically 4–5 mCi or 148–185 MBq) is approximately 50% lower, and the physical decay approximately twice as fast (^68^Ga t_½_ = 68 min). This typically results in > 60% lower true coincidence events for ^68^Ga-DOTATATE PET compared to ^18^F-FDG PET imaging [28]. The physical characteristics of ^68^Ga compared to ^18^F are also inferior due to partial volume effect, resulting in lower peak detected activity. The combination of these factors contributes to higher image noise and lower signal-to-noise ratio (SNR) of ^68^Ga compared to ^18^F in reconstructed tomographic images [28].

Normal ^68^Ga-DOTATATE PET hepatic uptake is also likely influenced by individual patient tumor burden, or the “sink effect” [30]. These differences in liver ^68^Ga-DOTATATE PET uptake, compared to FDG liver uptake, support the rationale for use of individual patient specific normal liver uptake and the variability metric in image noise (i.e., standard deviation of the background) to define a relatively high threshold level for hepatic lesion identification. Lesions detected by this method were confirmed as positive when compared to the clinically reported studies.

Although the precise reason for false positives and false negatives in DL algorithms is difficult to ascertain, we subjectively examined the discordance between the predicted and annotated gold standard (i.e., semi-automated data annotation method) and their relationships to their slice locations and relative intensity of uptake. The most common false positives (6/12; 50%) occurred where the predicted transaxial slice of a lesion was located at the edge of a true lesion, scored above the PERCIST threshold, but was discordant with the annotated gold standard which was located on an adjacent slice (i.e., the gradient edge detection rule determined that the boundary was located in an adjacent transaxial slice). With each slice treated as a discrete entity in the 2D CNN, the transaxial slice with high activity may be predicted by the model, but was not considered true positive by the annotated gold standard. The gradient edge detection determined that the boundary edge was in the adjacent slice.

Another discordance may result if the voxels are below the PERCIST criteria threshold for inclusion as true positive in the gold standard annotated dataset. These regions may not be labeled as true lesions in the gold standard training dataset, although visually they could be considered positive. Several false positives generated by the model are hypothesized due to this effect (4/12; 33%).

False positive thought due to misregistration between PET and CT occurred in only two instances (2/12; 17%) and represented the smallest potential source of error. Finally, the use of a voxel noise filter was used to reduce small regions of false positives because relatively high image noise could have a similar appearance to true hepatic lesions.

False negatives were subjectively thought to be primarily attributable to low uptake in parts of the lesion which were not detected on a specific slice (35/59; 59%), but located adjacent to a true lesion. The next most common category could be due to low or modest lesion uptake (21/59; 36%), not located adjacent to a detected true lesion. A small number of undetected true lesions, despite relatively high uptake, were not predicted and classified as unknown cause for missed lesions (3/59; 5%).

The primary limitation of this study is the relatively modest sample size for the development of the CNN. A 2D dataset was therefore implemented to obtain 233 unique tumor regions. As a single site feasibility study, our dataset size is of modest size due to the relatively recent introduction of ^68^Ga-DOTATATE PET, and the limited number of indications compared to FDG PET. This 2D algorithm does not incorporate the information from the adjacent transaxial images. A larger sample size and use of a 3D CNN could result in better algorithm performance. The second limitation arises from high noise images, and high normal background in liver ^68^Ga-DOTATATE studies. Image noise can be partially reduced by longer image acquisition times, or by using data from modern high sensitivity digital PET cameras. Most centers, however, have practical imaging protocols designed to balance image quality and efficiency, and older generation analog PET cameras. These data from this study are obtained from an analog PET system, which is typical of retrospectively collected real-world data.

Finally, our study also limited the task to detection of a relatively modest number of fewer than 10 non-confluent lesions with excluded patients with high volume liver disease. Patients with intrinsic liver disease including cirrhosis or steatosis were also excluded. The first restriction simplified the type of lesions to detect by excluding large confluent lesions with highly variable morphology; however, nuclear medicine physicians can readily recognize this pattern. The second restriction reduced the potential for variability in the background to avoid unknown effects of diseased liver uptake which could affect the threshold value used by the modified PERCIST criteria. In the future, larger datasets may be developed to identify lesions specifically within these subpopulations.

## Conclusion

This single-center pilot study demonstrates the feasibility of automated deep learning detection of hepatic lesions in ^68^Ga-DOTATATE PET/CT. Our use case supports the detection of clinically relevant lesions with high specificity. Ongoing improvements in data annotation methods, increasing sample sizes and improvements in algorithm training methods are anticipated to further improve detection performance.

## Supplementary Information

**Additional file 1.** Detailed information is supplied for performance metrics, the loss function for lesion detection, and model implementation.

## Data Availability

The datasets used and/or analyzed during the current study are available from the corresponding author on reasonable request.

## Abbreviations

CNN: Convolutional neural network
FDG: Fluorodeoxyglucose
GEP-NET: Gastroenteropancreatic neuroendocrine tumor
PPV: Positive predictive value
PR AUC: Precision–recall area under the curve

## Code availability

Questions regarding the code implemented in this work can be addressed to the corresponding author.
