# Supplementary material for: Automated liver lesion detection in 68Ga DOTATATE PET/CT using a deep fully convolutional neural network
Source: EJNMMI Res. 2021 Oct 2;11:98. doi: 10.1186/s13550-021-00839-x (PMC8487415; doi:10.1186/s13550-021-00839-x)
Supplement: Supplementary file 1 — Additional file 1. Detailed information is supplied for performance metrics, the loss function for lesion detection, and model implementation. [file 13550_2021_839_MOESM1_ESM.docx]

**SUPPLEMENTAL MATERIAL**

**Performance Metrics**

Although less intuitive to interpret than sensitivity/specificity, F_1_ score was a more appropriate metric given the imbalanced dataset. A tumor being present in any given slice is a relatively low probability. Weighing positives and negatives equally, and predicting every slice as having no lesions, for example, could generate a high score but be a specious representation of true performance. F_1_ score instead is the weighted average of precision (PPV) and recall (sensitivity) and gives a better representation of how the model actually performs.

**Loss Function for Lesion Detection**

Dice loss was selected as it is one of the most commonly used loss functions in deep learning involving medical images and handles class imbalance well. The Sørensen–Dice coefficient is defined as 2 times the overlap of the prediction and ground truth over the total number of pixels in both. The loss function used for training is then defined as 1 minus the Sørensen–Dice coefficient [1]. The dice loss function for lesion detection is defined as

$\mathcal{L}_{DICE}\left( y_{n},\hat{y}_{n} \right)= 1 - 2\frac{\left| y_{n}\cap\hat{y}_{n} \right|}{\left| y_{n} \right|+\left| \hat{y}_{n} \right|} = 1 - 2\frac{\sum_{n=1}^{N} y_{n}\hat{y}_{n} + \delta}{\sum_{n=1}^{N} y_{n} + \hat{y}_{n} + \delta}$

Where $y_{n}$ represents the ground truth, $\hat{y}_{n}$represents the predictions and $\delta$ is a small constant to ensure loss function stability and avoid division by 0.

However, dice loss has some important limitations that are relevant to our study. Insensitivity to the distance of non-overlapping regions is the first. If a prediction and ground truth do not overlap, the dice loss will be the same regardless of the proximity of the two shapes [2]. This means that a prediction cannot be brought closer to the ground truth if there is no initial overlap. The second is greater sensitivity to small lesions versus large lesions [3, 4]. One discordant pixel between the ground truth and prediction incurs a relatively small penalty in a large lesion. In smaller lesions, however, that pixel may constitute a substantial portion of the lesion area and the penalty will be much higher despite the same pixel missed in both cases.

To address the limitations of using the dice loss only, binary cross entropy was also incorporated into the model training. Compared to dice loss, binary cross entropy provides smoother training but might have difficulty in handling class imbalanced datasets [5]. The binary cross entropy loss function calculated as

$$\mathcal{L}_{BCE}\left( y_{n},\hat{y}_{n} \right)=-\frac{1}{N}\sum_{n=1}^{N} \left( \omega y_{n}\log(\hat{y}_{n} + \varepsilon)+ (1- y_{n})\log(1- \hat{y}_{n}+ \varepsilon) \right)$$

Where $y_{n}$ represents the ground truth, $\hat{y}_{n}$represents the predictions, $\omega$ is a constant for lesion weight to account for class imbalance and $\varepsilon$ is a constant to ensure loss function stability and avoid multiplication by zero.

Neither dice nor binary cross entropy loss performed particularly well alone, so a combo loss function was implemented. The combo loss used is a weighted linear combination of the dice and binary cross entropy loss functions. The combination of binary cross entropy and dice losses allows for some diversity in the loss with stability on an unbalanced dataset and has been shown to outperform both dice and binary cross entropy alone [5]. The BCE-DICE loss is calculated as

$$\mathcal{L}_{COMBO}\left( y_{n},\hat{y}_{n} \right)=\alpha\left( -\frac{1}{N}\sum_{n=1}^{N} \left( \omega y_{n}\log(\hat{y}_{n} + \varepsilon)+ (1- y_{n})\log(1- \hat{y}_{n}+ \varepsilon) \right) \right) + \beta\left( 1 - 2\frac{\sum_{n=1}^{N} y_{n}\hat{y}_{n} + \delta}{\sum_{n=1}^{N} y_{n} + \hat{y}_{n} + \delta} \right)$$

Where$y_{n}$, $\hat{y}_{n}$, $\delta$, $\omega$ and $\varepsilon$ are all the same as above and $\alpha$ and $\beta$ represent the weights of the BCE and Dice loss functions, respectively.

**Implementation Details**

The model was trained using stochastic gradient descent with Nesterov momentum [6]. Parameters were set as: learning rate = 5 × 10^-5^, momentum = 0.99, batch size = 8 and number of iterations = 10^5^. For the loss function, the variables were set as: $\alpha$ = 6, $\beta$ = 1, $\delta$ = 10^-6^, $\omega$ = 5 and $\varepsilon$ = 10^-10^. Training was stopped if performance on the validation set did not improve for 2 × 10^4^ iterations. The model was implemented with PyTorch [7] and trained and tested on a machine with 4.0 GHz Intel® Xeon® CPU and Nvidia Quadro RTX 5000 GPU. To enlarge the training dataset, data augmentation including shifting, mirroring and random rotation up between -180 and 180 degrees was used.

1. Milletari F, Navab N, Ahmadi S, editors. V-Net: Fully Convolutional Neural Networks for Volumetric Medical Image Segmentation. 2016 Fourth International Conference on 3D Vision (3DV); 2016 25-28 Oct. 2016.

2. Rezatofighi H, Tsoi N, Gwak J, Sadeghian A, Reid I, Savarese S. Generalized Intersection over Union: A Metric and A Loss for Bounding Box Regression2019 February 01, 2019:[arXiv:1902.09630 p.]. Available from: https://ui.adsabs.harvard.edu/abs/2019arXiv190209630R.

3. Guizard N, Coupé P, Fonov VS, Manjón JV, Arnold DL, Collins DL. Rotation-invariant multi-contrast non-local means for MS lesion segmentation. NeuroImage: Clinical. 2015;8:376-89. doi: https://doi.org/10.1016/j.nicl.2015.05.001.

4. Harmouche R, Subbanna NK, Collins DL, Arnold DL, Arbel T. Probabilistic Multiple Sclerosis Lesion Classification Based on Modeling Regional Intensity Variability and Local Neighborhood Information. IEEE Transactions on Biomedical Engineering. 2015;62(5):1281-92. doi: 10.1109/TBME.2014.2385635.

5. Taghanaki SA, Zheng Y, Kevin Zhou S, Georgescu B, Sharma P, Xu D, et al. Combo loss: Handling input and output imbalance in multi-organ segmentation. Comput Med Imaging Graph. 2019;75:24-33. Epub 2019/05/28. doi: 10.1016/j.compmedimag.2019.04.005. PubMed PMID: 31129477.

6. Sutskever I, Martens J, Dahl G, Hinton G. On the importance of initialization and momentum in deep learning. In: Sanjoy D, David M, editors. Proceedings of the 30th International Conference on Machine Learning; Proceedings of Machine Learning Research: PMLR; 2013. p. 1139--47.

7. Available: https://githubcom/pytorch. 2021, [online].
